# From dry-land to the water: training and testing practices of strength and conditioning coaches in high level French sprint swimmers

**DOI:** 10.3389/fspor.2023.1338856

**Published:** 2024-01-12

**Authors:** Yannis Raineteau, Robin Pla, Benoit Bideau, Nicolas Bideau, Guillaume Nicolas

**Affiliations:** ^1^ M2S Laboratory—Laboratoire Mouvement Sport Santé, Université Rennes 2, Rennes, France; ^2^ Optimization Service, Fédération Française de Natation, Clichy, France; ^3^ MIMETIC-Team, INRIA Rennes Bretagne Atlantique, Rennes, France

**Keywords:** transfer, competitive swimming, survey, specificity, exercises

## Abstract

**Introduction:**

The aim of this study was to explore training and testing practices from Strength & Conditioning (S&C) coaches who manage groups of high-level French swimmers in elite training centers. The transfer of abilities from dry-land to *in situ* condition was also investigated.

**Methods:**

24 French S&C coaches completed a survey via an online platform. Frequency analyses were made for quantitative and qualitative responses, the level of significance set for this study was *p* ≤ 0.05.

**Results:**

Core stability, Strength & Power were the three most targeted qualities. Core strengthening in all its forms, Bench Press & Squat were the three most prescribed exercises. 79% of S&C coaches adapted exercises according to different parameters. Most of the coaches indicated that dry-land S&C sessions were preferentially placed before in-water sessions. Very varied exercises were used in-water to make the transfer from dry-land more effective. 87% of participants monitored the training load and 38% assessed the force and velocity parameters for some S&C exercises.

**Discussion:**

Dry-land training practices of S&C coaches were mostly in line with scientific recommendations. In the light of results of the questionnaire, it would appear that testing procedures might be a key issue for transferring qualities from dry-land to *in situ*.

## 1 Introduction

Strength and conditioning (S&C) for swimmers is defined as the physical and physiological development of swimmers provided either by the swimming coach or by a specialized S&C coach using mainly dry-land exercises. The role of S&C is viewed as a way to prevent injury, strengthen the athlete's body in a general way and specifically improve performance in the water (1, 2). Swimming is a repetitive and cyclical sport with unique physiological and biomechanical demands due to the large variety of racing distances spread across multiple swimming stroke techniques (3), which may lead to specific injuries (4). The most affected body areas are the shoulder, knee, and lower back (5). Strength, posture, and mobility exercises can help stabilize joints and compensate for muscle deficits in some agonist/antagonist pairs (6).

As regards to swimming performance, one of the difficulties regarding S&C is to take the features of the aquatic environment into account, as it implies specific constraints in comparison to the terrestrial environment (7). More particularly: 1. Swimmers are in a prone position; 2. Both arms and legs are used actively for propulsion; 3. The propulsive forces produced by the athlete are applied to a moving medium. Hence, only a part of the total mechanical power output is used beneficially to overcome body drag, the other being dissipated giving water un-useful kinetic energy; 4. Large 3D sculling movements are created with fluctuations in speed and orientation, depending on the four strokes; 5. Transitional phases (start, turn, and underwater phases) play a significant role in swimming performance. Another difficulty lies in the fact that the transfer between S&C in dry-land condition and the efficiency of propulsion in a swimming situation is not obvious. Therefore, the intervention of the S&C coach with swimmers may be tricky. From a practical point of view, there are still many questions about the criteria to provide guidance concerning the training contents in S&C regarding modalities, swimming stroke specialty, level of expertise, upper and lower body differentiation, and muscle contraction regimes (7). While there is almost unanimous agreement on the positive links between different dryland strength and power abilities and swimming performance, especially for sprint event (8–10), the development methods of these abilities remain a controversial topic. Most previous studies have shown positive effects of different types of strength work such as maximal strength (11–13), hypertrophy (14–17), strength endurance (18, 19) or explosivity (20–22) on performance. More rarely, some studies showed negative effects of the development of these abilities on performance. For example, some authors observed a decrease in 25-yard swimming speed after 8 weeks of dryland hypertrophy work in collegiate swimmers (23). Similarly, other authors also observed a decrease in 50 m swimming speed after dryland maximal strength work of upper limbs on master swimmers (24). The results of these studies raise the problem of comparing swimmers of different levels and with different previous experience in S&C. These differences in results could also be explained by the various parameters that may affect the transfer of strength and power qualities into swimming (dryland work modalities, combination of dry land and swimming workouts, training periodization, etc.). As regard to the combination of dryland and swimming workouts, some authors evaluated the effects of resistance workouts in the water (17, 25) or power workout on a swim bench ergometer (16, 26) and showed significant improvements in performance.

More generally, the literature on the S&C practices in swimming remains limited, and more specifically as regard to the transfer from dry-land to in-water condition, the testing and the link between S&C and performance. Despite the few discrepancies that may still exist as regards to benefits of S&C programs to performance, S&C remains an increasingly important part of swimmer preparation. Usually, the preparation of an Olympic swimmer is schematically composed of three or four sessions out of the water, based essentially on hypertrophy (8 to 10 repetitions at 65%–80% of 1RM) or on maximum strength (less than 6 repetition at 85%–100% of 1RM) (27).

In order to understand the role of S&C on performance, a couple of studies reported different modalities of field practices, usually using questionnaires. Indeed, questionnaires are a good way of surveying practices on the field. This method of collecting information was successfully used to identify S&C work in several sports such as soccer (28), football (29), or basketball (30). Whereas generic questionnaires were used for these latter sports that rely on similar physical qualities, the use of a pre-established questionnaire in swimming is not possible due to the specificities of the fluid medium mentioned above. Indeed, specific questionnaires have previously been used in swimming to identify on-field warm-up techniques (31), training load monitoring (32), injury surveillance (4), performance analysis (33) or specific training points such as underwater kicks (34). In the context of S&C, some studies investigated S&C practices on competitive swimmers (35–38). More specifically, some studies (36, 38) concomitantly evaluated S&C practices and the swimming training part, but without connection between the two fundamental training conditions. More recently, some authors (37) also investigated the S&C practices in swimmers of different levels (from regional to Elite level) and countries. This study thus highlighted key points in swimming S&C practices such as the predominant use of strength workout and the different dry-land resistance training practices (warm-up, circuit training, traditional resistance training and plyometrics). They also identified pull-up and squat as the most popular dry-land resistance training exercises. However, as underlined by the authors, S&C coaches' practices regarding the specificity and the transfer of resistance training exercises to swimming performance remains a topic which needs to be investigated.

Whereas most studies were focused on the S&C training part, the evaluation of the quality of this dry-land to *in situ* transfer may be improved by identifying other objective indicators that relate to testing practices. In this aim, a first approach lies in the implementation of a specific questionnaire that: 1. Explores the S&C coaches' practices that link dry-land workout with swimming training, 2. Includes training but also a testing part [i.e., evaluating swimmers via dynamic parameters both in dry-land and in water conditions (9)]. In this study, we developed a specific survey that addressed these issues and that included specific topics such as specificity of the strokes, strength workout modalities, limbs differentiation that may affect differently the dry-land to *in situ* transfer (39).

## 2 Methods

### 2.1 Experimental approach to the problem

The design of this survey was based on the above-mentioned studies (4, 32, 34, 37). It included specific questions or items devoted to training in dry-land but also in the water conditions, and items dedicated to the tools and methodologies used to evaluate the transfer of qualities from dry-land to *in situ* conditions. The survey was edited on LimeSurvey, a statistical online survey software which complies with the General Data Protection Regulation (GDPR) confidentiality standards. It was composed of 54 questions, divided in 5 categories associated with both testing and training: 1. S&C coaches' personal information including experience and education, 2. Description of prescribed dry-land workout, 3. Self-Analysis on their S&C intervention, 4. Characterization of dry-land to in-water transferring workout, and 5. Methodologies for testing in both dry-land and *in situ* conditions. Prior to sending the final version of the survey questionnaire, a pilot testing was conducted by 2 national S&C coaches, 2 members of the Performance Optimization department of the French Swimming Federation and 2 researchers implicated in this study, which enabled to refine the survey conception. Each part of the questionnaire was designed to investigate the dry-land training to *in situ* practices in order to examine more specifically the transfer process. This study conforms to the Checklist for Reporting of Internet Surveys (CHERRIES) (40). A copy of the survey (Supplementary Material A) as well as the CHERRIES checklist (Supplementary Material B) are available online.

### 2.2 Subjects

Inclusion criteria for S&C coaches answering the survey consisted of working in the French Swimming Federation certified training centers such as National Training Centers, High Level Training Center, or Development Training Center, whereas some swimmers supervised by the S&C coach had to perform at least at a national level of performance. These inclusion criteria corresponded to thirty-three S&C coaches (population size). The sample size was calculated using a confidence interval of 95%, a margin of error of 5% and assuming that 5% of S&C coaches in French swimming were working on high-level swimmers (population proportion). Of the thirty-three S&C coaches contacted, twenty-four (72.7%, *n* = 21 men, *n* = 3 women) answered the complete survey. Nine of them were also in charge of the in-water training. Average experience in the job of S&C coach was 7.1 ± 5.5 years. Each participant had to agree to an online informed consent form to participate in the research. The study was approved by the Ethics Committee of the university to which the laboratory where the study was conducted is attached (Approval code: 2023-004).

### 2.3 Procedures

The survey was transmitted via a private mailing list. Email addresses were collected by the French Swimming Federation. In the first email of transmission, the purpose and the interests of the research were presented. Subsequently, further reminder emails were sent and a reminder from the federation was made. The survey was closed 2 months after it was opened for responses. Some demographic information was requested, such as respondent's first and last names as well as the name of the team the respondent belongs to. The data collected was then pseudonymized and stored in a protected digital space. IP address was used to avoid answers duplication. Three respondents did not complete the whole survey and were therefore excluded from the present analysis.

### 2.4 Statistical analysis

A first analysis was made using the responses to quantitative questions and then a complementary analysis was made using qualitative open-ended and closed questions. All the processing was performed using Microsoft Excel (Microsoft Corporation, Redmond, USA). Frequency Analysis was made on 26 quantitative and qualitative questions. As regards to the part devoted to self-analysis of their S&C intervention, coaches were asked to assign scores from 1 to 4 the arguments they felt most closely matching their views. Numbers were associated with the order (1 for the most meaningful and 4 for the furthest from their views) and the mean value for each argument was calculated which helped to highlight the arguments.

## 3 Results

### 3.1 Coach characteristics

88% of participants had an academic background (university degree in S&C or in Sport Science). 43% of them also had a professional degree or state certificate in sport coaching. 46% (*n* = 11) of them had a specific degree in S&C whereas others graduated in the field of training in swimming. Most S&C coaches (88%, *n* = 21) trained groups that included sprinters. Among the 15 participants whose sole function lies in S&C coach, 93% (*n* = 14) were also involved in other activities than swimming. This proportion was much lower for the 9 participants who were also responsible for the in-water training (33%, *n* = 3). Other activities supervised by the participants that were mostly reported were: handball (*n* = 5), tennis (*n* = 4), rugby (*n* = 4) and football (*n* = 3).

### 3.2 Strength and conditioning practices

83% of coaches included three or more S&C sessions per week. The S&C sessions represented at least three hours of workout for 92% of the respondents to the survey.

**Figure 1:**
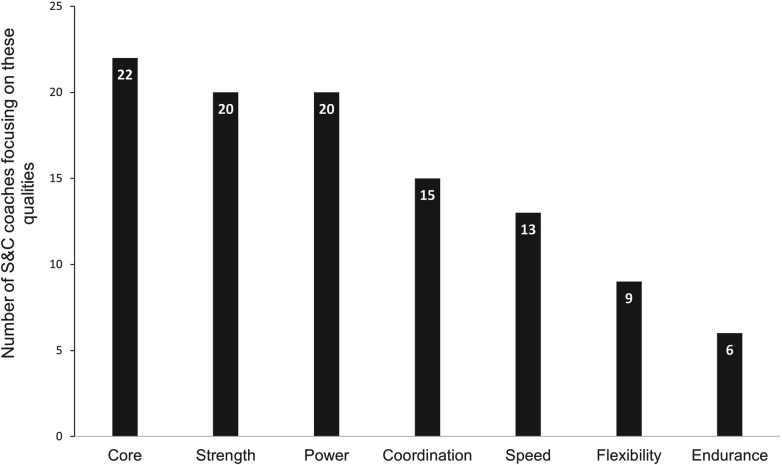
Physical abilities mostly intended by S&C coaches in swimming.

Figure 1 shows that the core stability, the strength, and the power were the mostly intended qualities by S&C coaches. Nevertheless, all the responders specified that all qualities were addressed during different training periods of the season.

Other results showed that as many S&C coaches differentiated (*n* = 12) the qualities between lower limbs work and upper limbs work as those who did not (*n* = 12). For the S&C coaches who differentiated these qualities, the argument was that the lower limbs are more related to strength and explosiveness qualities whereas endurance and hypertrophy are prohibited because (sic): “strength, endurance and hypertrophy are not necessary for lower limbs since their use in swimming is mainly related to start and turns, which rather requires plyometric qualities” and because (sic) “the lower limbs can sustain much heavier loads than the upper limbs without risk of injury”. 58% of the S&C coaches stated that they worked more on the upper limbs than the lower limbs, while only one coach worked mainly on the lower limbs. The others reported working both upper and lower limbs to an equal extent.

The different abilities were periodized differently, following a whole season scale or a macrocycle scale. On a whole season scale, hypertrophy (*n* = 10), and strength endurance (*n* = 7), seemed to be the two most prescribed training modalities, especially during the beginning of the season. Maximal strength (*n* = 4), maximal power (*n* = 4), and maximal speed (*n* = 4) were the most cited types of workouts that were included continuously in the season. On a macro-cycle scale, training plans seemed to start from maximal strength and evolve towards maximal speed as the targeted competition approached. Indeed, 11 coaches indicated that maximal strength workouts were prescribed during development phases. Coaches also indicated that maximal power workouts were prescribed after maximal strength (*n* = 9) or just before the targeted competition (*n* = 6), and finally that maximal speed workouts were prescribed just before the targeted competition (*n* = 12). This result can be balanced by the fact that for middle-distance swimmers, strength endurance was the main ability prescribed just before the targeted competition.

Regarding the workouts prescribed for maximal strength development, main types of muscle actions were the concentric mode (*n* = 22; 92%), then eccentric mode (*n* = 16; 67%), stato-dynamic mode (*n* = 16; 67%) and finally isometric mode (*n* = 11; 46%). Average number of sets was between 3 and 4 (min: 1; max: 6), average number of repetitions between 3 and 4 (min: 1; max: 6), average load 88,8% of 1RM or RPE 7 (min: 80%; max: 110%) and average time of recovery period 3 min 06 s (min: 45 s; max: five minutes).

Regarding the workouts prescribed for maximal power development, the main types of muscle actions were the concentric mode (*n* = 22; 92%), and then eccentric mode (*n* = 17; 71%). Average number of sets was between 3 and 4 (min: 3; max: 8), average number of repetitions between 6 and 7 (min: 2; max: 15), average load 58.8% of 1RM (min: 30%; max: 85%) and average time of recovery period 2 min and 42 s (min: 30 s; max: 5 min).

**Figure 2:**
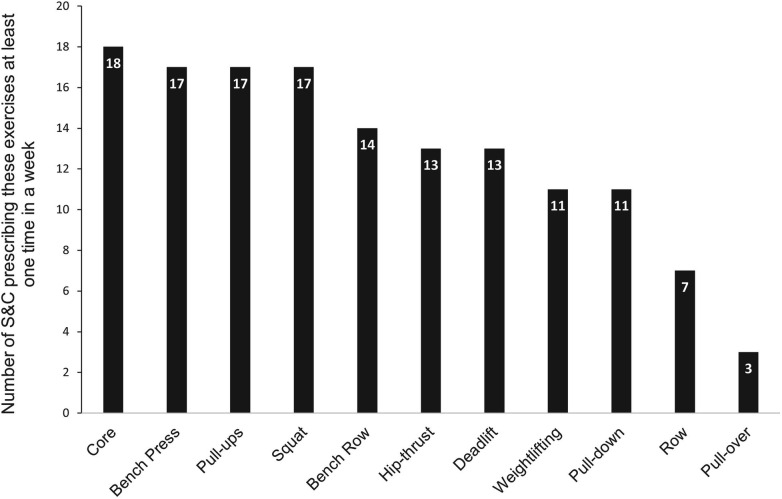
Strength exercises mostly intended by S&C coaches.

Figure 2 illustrates the most prescribed strength exercises by S&C coaches during the season.

However, responses mentioned in Figure 2 were tempered by the fact that for 79% of the responders, a given strength exercise (core, bench press, etc.) may undergo potential adaptations according to different parameters such as:

- Muscle mass involved in the different strokes (*n* = 9): Sumo deadlift or full squat for breaststrokers (*n* = 5), standing pull-down for butterfliers (*n* = 1), dissociated work (e.g., alternated dumbbell shoulder press) for alternated strokes (*n* = 1),
- Periodization in the season (*n* = 2),
- Injuries history (reathletization and prevention, *n* = 1),
- Swimming physiological or biomechanical deficits (*n* = 1).

**Figure 3:**
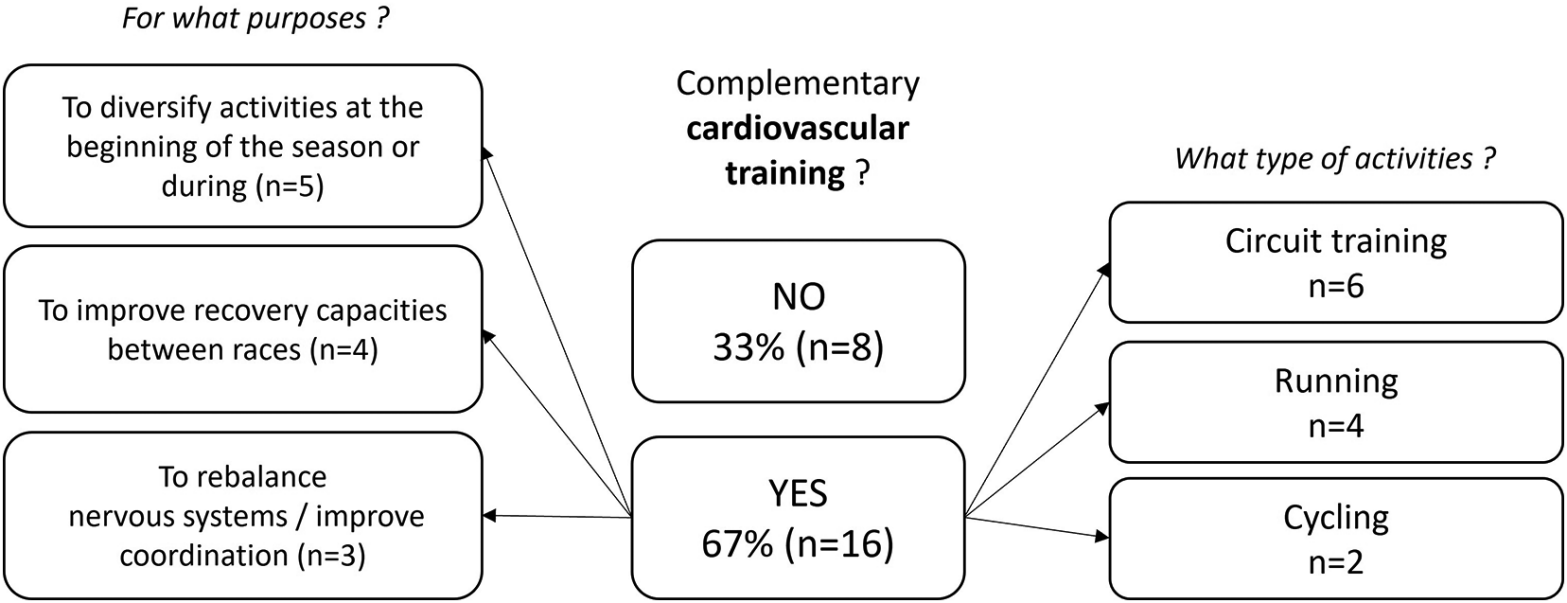
Uses, practices and justifications of complementary cardiovascular training as reported by 24 responders.

**Figure 4:**
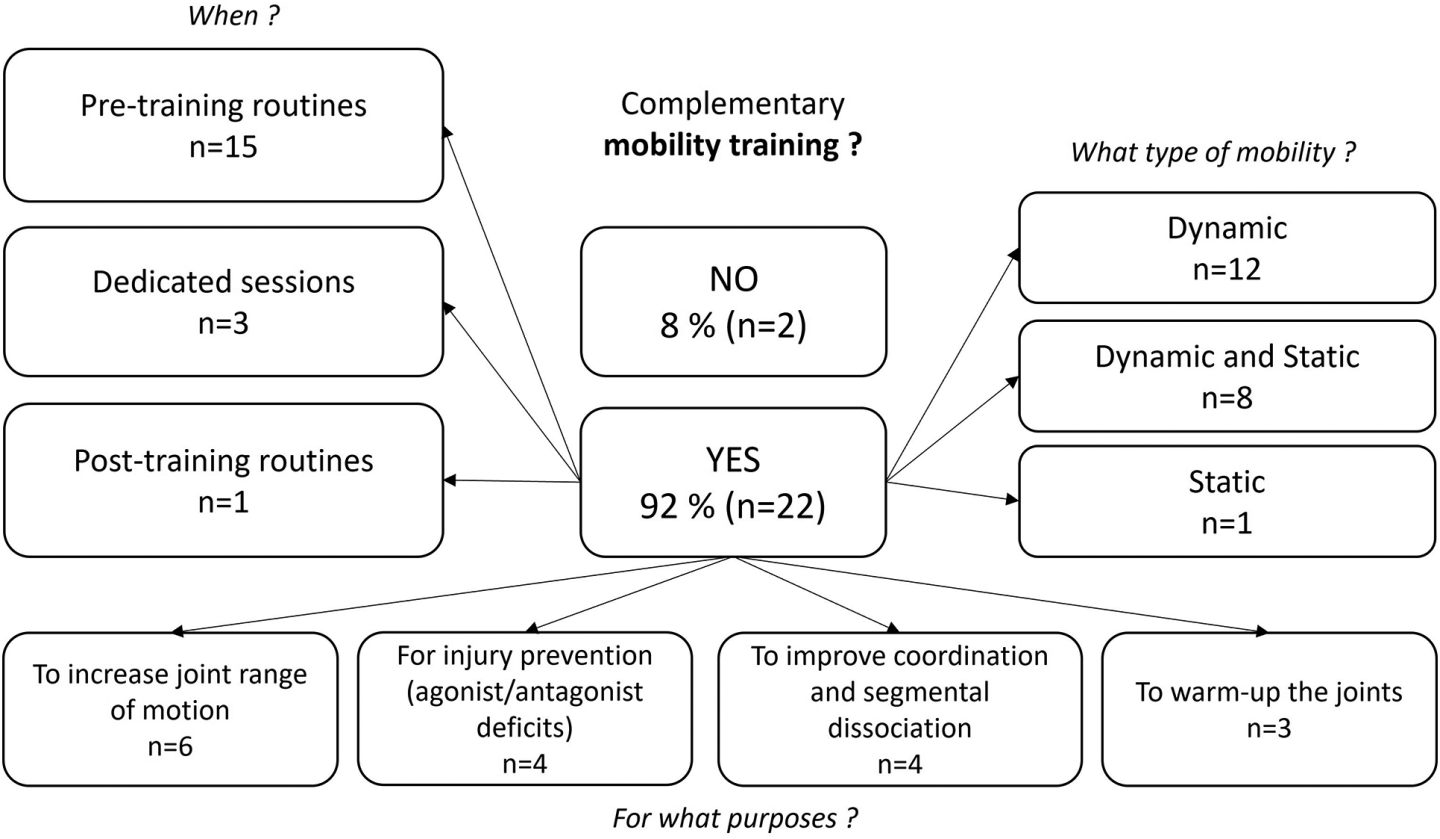
Uses, practices and justifications of complementary mobility training as reported by 24 responders.

In addition to the strength exercises, cardiovascular & mobility (i.e., increasing the joint range of motion) training were introduced (Figures 3, 4). Complementary cardiovascular training was mainly achieved in the form of circuit training in order to diversify activities at the beginning or during the season (*n* = 5), with the aim of improving recovery capacities (*n* = 4) or improving coordination (*n* = 3). Figure 4 illustrates the practices and justifications of complementary mobility training as reported by 92% of the responders.

### 3.3 Self-analysis on their S&C intervention

**Figure 5:**
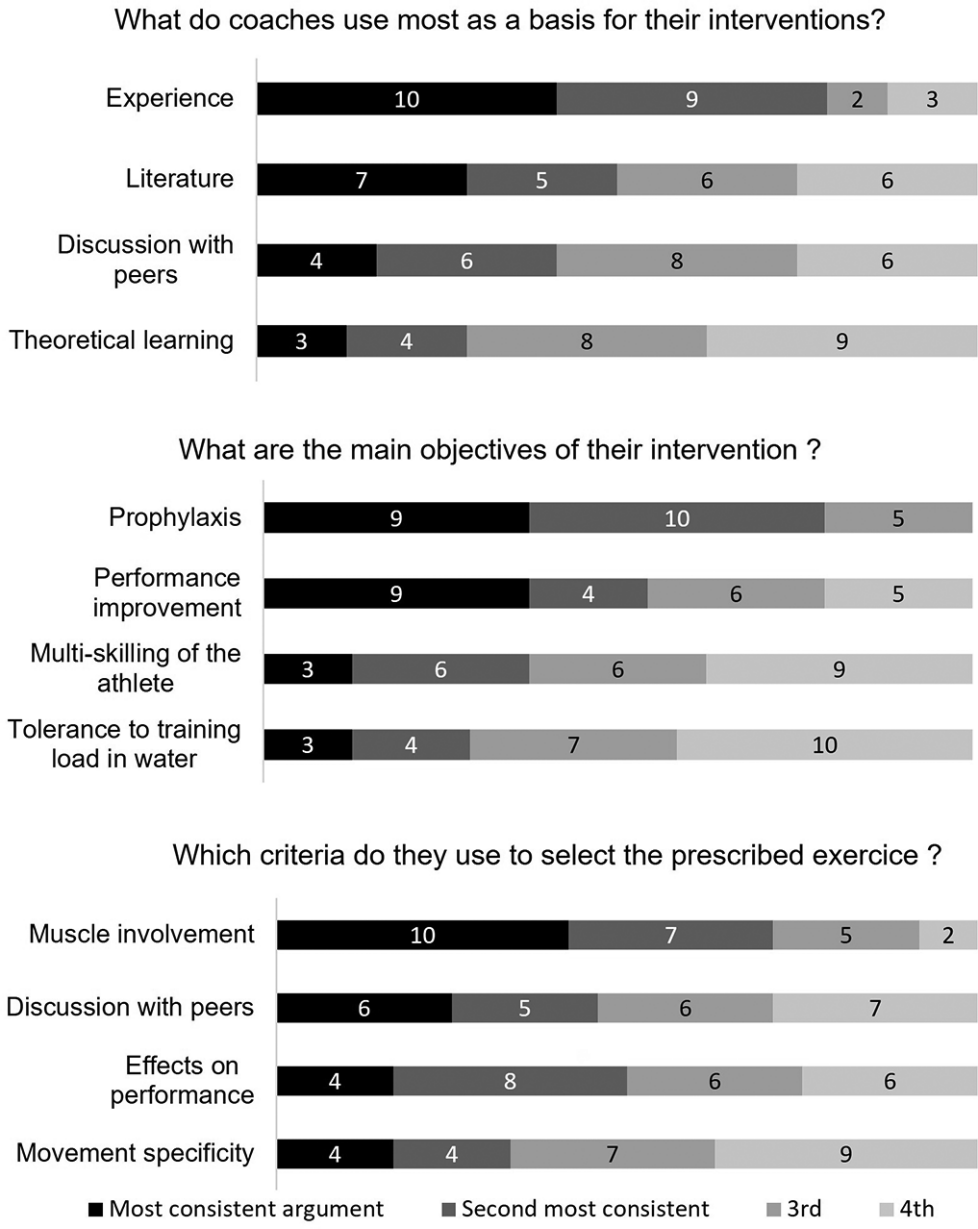
S&C coaches’ perception about the workout performed. Each responder was asked to rank from 1 to 4 the rationale that justifies his intervention on the field. For example, among the 24 responders, 10 of them may have provided “experience” as the most meaningful argument that guides their intervention, 9 coaches may have provided “experience” as the second argument, and so on.

This part of the survey was supposed to provide insights on how S&C coaches set up their sessions. Figure 5 illustrates the S&C coaches' perception about the workout performed with regard to 3 issues: Rationale for their intervention, objectives of their intervention, rationale behind the choice of strengthening exercises. The order remained the same whether the participants were only S&C coaches or also swimming coaches.

### 3.4 Dry-land to in-water transfer practices

Communication between staff members was cited as the most crucial factor to optimize the transfer from dry-land to in-water (*n* = 14). These responders reported that staff members have first to agree on priorities concerning both dry-land and *in situ* conditions. Consistency and congruence in planning of S&C and in-water training were the next most mentioned actions by the participants (*n* = 10). Finally, sequence of *in situ* and dry-land sessions (S&C preceding or not in water training) on a one-day scale also appeared to be important for the S&C coaches regarding the optimization of the transfer (*n* = 8). Regarding this latter aspect, coaches reported that swimmers had a S&C session and a swimming session on the same day between 3 and 4 times per week on average (mean: 3.2, min: 1, max: 5). These two sessions were consecutive between 2 and 3 times per week on average (mean: 2.6, min: 1, max: 5). For the consecutive sessions, coaches reported that the S&C sessions were mainly placed before swimming (50%; *n* = 12). It was also reported that the two orders were equally represented (33%; *n* = 8), while it was less common that the swimming session was placed before the dry-land session (17%; *n* = 4). These orders are mainly linked to time constraints (54%; *n* = 13) but are sometimes a deliberate choice of the coach (42%; *n* = 13). When the orders were: “both equally” or “swimming session preceding S&C session”, it is less due to the choice of coaches (*n* = 1 and *n* = 1 respectively) than when the order is “S&C session preceding swimming session” (67%; *n* = 8).

**Figure 6:**
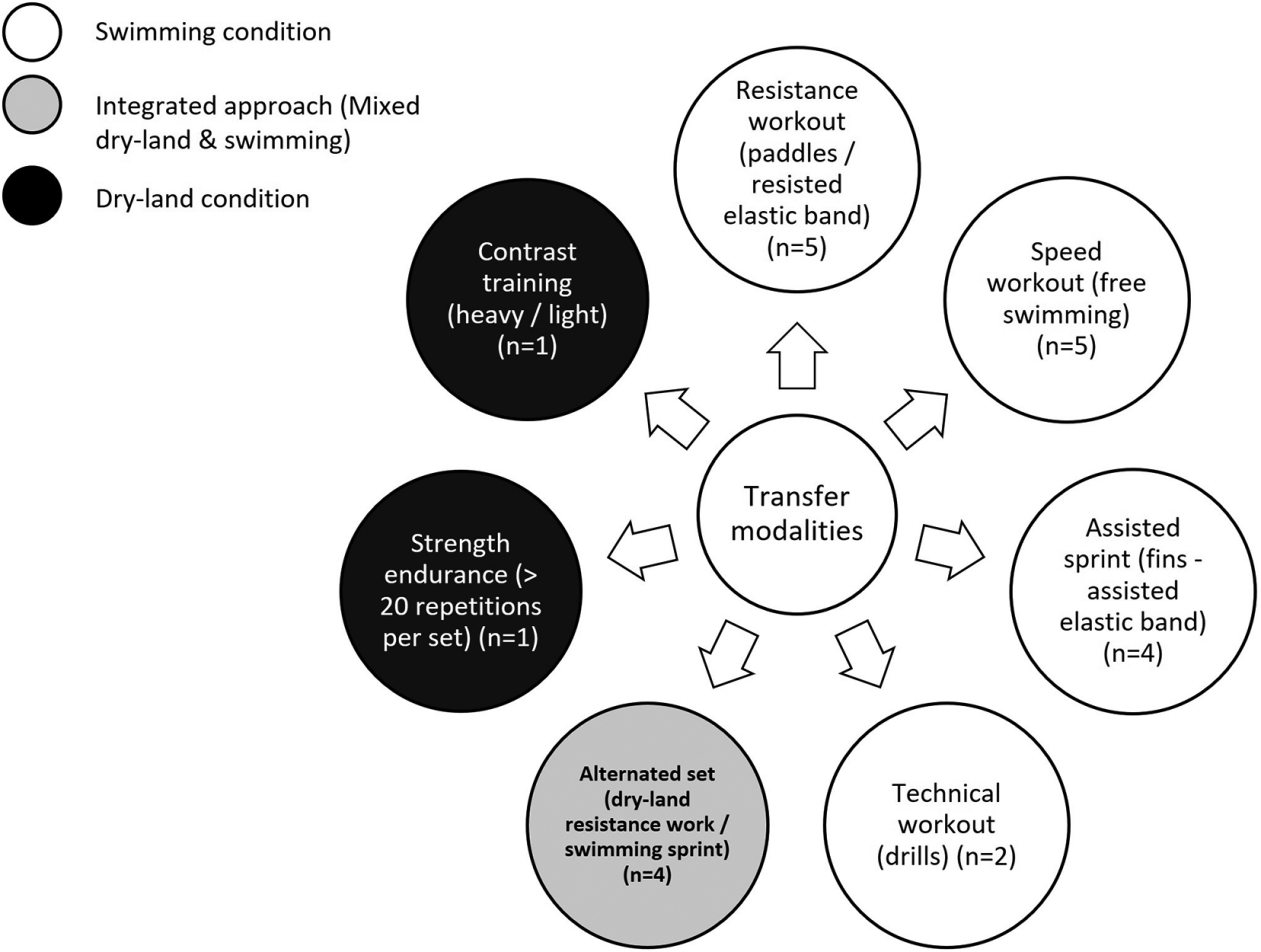
Intended modalities for transfer of the dry-land workout to the swimming stroke.

Figure 6 illustrates the intended modalities reported by S&C coaches that were supposed to improve the transfer from dry-land condition to the swimming stroke. Responders suggested that overspeed mostly occurs after *in situ* resistance workout for a load contrast. Integrated workouts (i.e., mixed dry-land and swimming workouts) were composed of “technical” (e.g., trying to improve distance per stroke; *n* = 2), “speed” workouts in water (*n* = 3), or explosiveness on start and turns phases (*n* = 1). The dry-land part of these integrated workouts was mainly focused on load contrast for maximal strength and maximal velocity development (*n* = 3), or maximal strength (*n* = 1).

79.2% of responders (*n* = 19), commented that parallel observation of progress in dry-land condition and in-water condition was carried out. Indicators used to observe this progression were mainly the swimming performance (*n* = 10) and swimming spatio-temporal parameters: distance per cycle, number of strokes and stroke frequency (*n* = 4). Physical integrity (absence of injury) and the ability of swimmers to complete all sessions were also highlighted as indicators linking S&C and swimming (*n* = 4). Testing and load progression in dry-land condition were also cited (*n* = 5).

### 3.5 Testing practices

**Figure 7:**
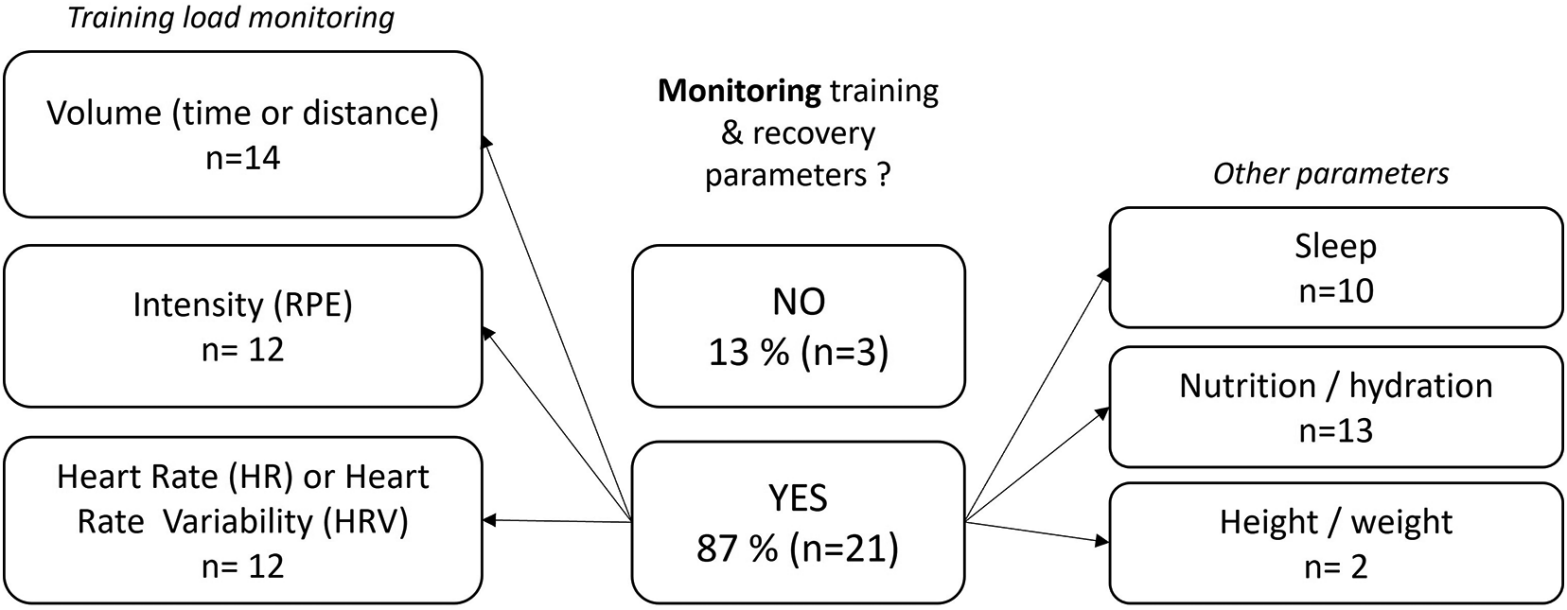
Summary of the main parameters monitored during training as reported by 24 responders.

Figure 7 shows which training or recovery parameters were monitored in every training group. Responders also indicated that dry-land S&C progress was assessed. It was reported to be achieved in several ways: through the load progression (*n* = 21), the evolution of the number of repetitions, or through the evolution of strength/speed/power parameters using digital devices (*n* = 11). The most commonly reported technologies were Ergometers such as SkiErg (Concept 2, Morrisville, VT, USA) or Wattbike (Wattbike LTD, Nottingham, UK). 38% (*n* = 9) of participants used numerical assessments of the force and of the speed of the barbell. Various technical solutions that measure the speed of the barbell were reported: Gymaware (Kinematic Performance Technology, Canberra, Australia) (*n* = 4), mobile applications such as MyJump or MyLift (*n* = 3), Beast (Beast Technologies, Brescia, Italy) (*n* = 2), VitruveFit (SPEED4LIFTS S.L., Mostoles, Madrid) (*n* = 1) and Myotest (Myotest SA, Sion, Switzerland) (*n* = 1). RPE was also reported to be a subjective but complementary means of evaluating progress in S&C.

Testing protocols were performed periodically, on average 4.75 ± 2.74 times per year. Responders also indicated that they carried out these testing protocols at key periods (during national team training courses, or at the beginning and end of each dry-land cycle) (*n* = 2).

## 4 Discussion

The aim of this study, based on a survey, was to explore the training and testing practices of S&C in high level swimming in France, specifically addressing the relationship between dry-land and *in situ* workout. The originality of this work lies in its global and systemic approach with the aim of understanding the links that may exist, and specifically the representations that coaches have of them, between dry-land S&C and performance in the water. 24 S&C coaches answered the survey, and 90% of them had an academic background with almost half of them having a professional degree or state certificate in sport coaching which attests to a level of knowledge in the field of S&C.

As regards to the objectives of their intervention, “Prophylaxis” was the most cited item, followed by the “improvement of swimming performance”, then “multi-skilling and improved versatility of the swimmers” and finally, “improved tolerance to the training load in water”. It may be surprising to note that prophylaxis was the primary objective cited even before performance improvement. This can be explained by the fact that the populations supervised by the coaches include swimmers whose physical maturation is not finalized, which justifies the prophylaxis argument.

### 4.1 Strength and conditioning practices

As regards to dry-land training, it was interesting to note that the most prescribed exercises are not necessarily the most specific in terms of force orientation as regards the ones achieved during swimming motion [e.g., Bench Press or Squat (41)]. This observation is consistent with the findings that coaches ultimately rely on the specificity of the exercise regarding the stroke to choose which exercise they will prescribe. Some recent research (41) contradicts this view, stating that force orientation specificity in S&C exercises is an important parameter for improvement of swimming performance. Nevertheless, other studies (1, 42) nuanced this idea, suggesting that classic strength training exercises (squat, bench press, lat pulldown) would remain the best way to develop strength out of water. These studies indeed suggest that dry-land workouts never correspond entirely to the stroke movement and that S&C coaches should therefore avoid falling into the trap of quasi-specificity.

However, a great majority of responders (79%) indicated that some of the exercises could be adapted according to various parameters. The main reported item was related to the muscle masses involved in the different strokes (*n* = 9) whereas other parameters were less mentioned: training period of the season (*n* = 2), injuries history (*n* = 1) or swimming physiological or biomechanical deficits (*n* = 1). The reason given by the coaches was that they were trying to solicit the most involved muscles as in the stroke is consistent with most previous studies from the literature. Indeed, the four strokes have been shown as kinematically different (43) but also from a neuromuscular point of view (44). For example, breaststroke has specific electromyographic features compared to other strokes (muscle level of recruitment and coordination) (45), especially concerning lower limb muscles activation (46). This specificity leads some coaches to use, for example, the sumo squat variation in order to activate in a more significant way the gluteal muscles, quadriceps and hamstrings which are particularly involved in this stroke (47).

In a more general way, upper limbs were stated to be reinforced more than the lower limbs (58% of the responders). This result is quite consistent with the idea that most of propulsion is generated by the arms' actions. In front crawl swimming, it was found (48, 49) that about 85% to 90% of propulsion is produced by the arms' movements. Nevertheless, leg's propulsion should not be disregarded in view of its impact on performance, both during swimming parts and during starts, turns and underwater undulatory swimming (UUS) which has become an essential and determining part of high-level performance (50, 51). Therefore, it is not surprising to observe for 50% of the responders a will to strengthen lower limb muscles with a plyometric component. In line with this, solicitation of trunk muscles is inherent to all four strokes (44), so it seems logical that core stability is commonly cited in the coach's survey, both as an exercise and as a quality to work on. Core stability would indeed be an important quality for the prevention of injuries because it would have an impact on scapular control and therefore on development of shoulder pain (52, 53). However, most studies that have investigated the effects of dry-land training on swimming performance or strength solely focused on exercises or qualities that involve only the upper or lower limbs without taking core into account (2).

More generally, maximal force and power development rely on different modalities. Maximal Strength training muscle actions were mainly represented by the use of concentric (92%), eccentric (67%) and stato-dynamic (67%) modes of contraction. Concentric and eccentric modes of contraction have been described as efficient training modalities to develop maximal strength (54). However, to our knowledge, there is no evidence of effectiveness of stato-dynamic training methods to enhance maximal strength. Further investigations need to be carried out, especially in swimming, to know if this is a reliable method to develop maximal strength. Other S&C modalities prescribed by S&C coaches in our study (3–4 sets, 3–4 repetitions, approximately at 90% of 1RM with 3 min and 6 s of rest in average) were in line with both scientific recommendations and S&C practices of Elite swimmers in other countries as for number of sets and repetitions (38, 55), chosen loads (56, 57) and rest intervals (58). Regarding maximal power training muscle actions, practices reported in the present study (i.e., concentric, and plyometric modes, 3–4 sets, 6–7 repetitions, around 60% of 1RM and with 2 min and 42 s of rest in average) are also in line with scientific recommendations and S&C practices of Elite swimmers in other countries.

In addition to the strength exercises, cardiovascular (i.e., running, cycling, extreme conditioning program training) & mobility (i.e., increasing the joint range) training were introduced, respectively for 67% and 92% of the responders. Many reasons were reported for the use of cardiovascular training in dry-land: to diversify activities (*n* = 5), to improve recovery capacities between races (*n* = 4), and to rebalance nervous systems/improve coordination (*n* = 3). As the aerobic training volume in-water is already significant (59), implementation of aerobic dry-land training would be questionable. However, some studies showed that maximal heart rate (HR) during running is higher than during swimming due to sport specific training adaptations (60). Thus, complementary activities such as running implemented during swimmers' preparation may be useful to reach higher HR intensities than the ones they can reach during swimming. S&C coaches mainly used mobility as a pre-training routine (*n* = 15), in dynamic (*n* = 12) or both dynamic and static way (*n* = 8). The main reasons reported for the use of mobility training were to increase joint range of motion (ROM) (*n* = 6), to prevent injury risk (*n* = 4), to improve coordination (*n* = 4) and to warm-up joints (*n* = 4). To our knowledge, no study could be found attesting to the effectiveness of this type of complementary work on injury incidence or performance in swimming. For these reasons, the practices of S&C coaches in this area may be influenced by practices found in other sports, but which may not correspond to the specific requirements of the aquatic environment in swimming.

### 4.2 Dry-land to in-water transfer practices

As previously underlined by the coaches, transfer from dry-land to in-water may also be an issue as regards to the objectives of training in swimming. It was cited to be accomplished using exclusive workouts in the water with various strategies (resistance workout using paddles or elastic band, *n* = 5; sprint workouts, *n* = 5; assisted sprint / overspeed workouts, *n* = 4). These trends are highlighted in a narrative review of S&C in swimming (1) who stated that power could be developed only after specific-neuronal activation. This assumes that the swimming velocity workout would better transfer the dry-land S&C improvements into the water. This assumption also appeared to be shared by some S&C coaches (*n* = 9) surveyed in the present study as speed and overspeed in water are often used to transfer these qualities. For coaches surveyed, resistance training in water also appeared as a key exercise for transferring strength from dry-land to the swimming stroke (*n* = 5). The authors of the above-mentioned review (1) were less in agreement with this use, highlighting the fact that the water would not offer enough resistance to develop sufficient force levels to induce adaptations. They therefore recommended, with this aim of transfer, to carry out the work in conditions that are as close as possible to free swimming, without equipment.

Beyond the training modalities, responders reported that staff members should first agree on priorities concerning both dry-land and *in situ* conditions. Consistency and congruence of S&C dry-land and in water training programs were the next most mentioned action by the participants (*n* = 10). This underlines, on the one hand, the difference between the will and the objectives defined by the coaches and, on the other hand, the “field” conditions (organizational etc.) offered to implement these objectives. Indeed, the order between S&C and swimming sessions was, in most cases, not a deliberate choice but rather due to time constraints.

Sequence of *in situ* and dry-land sessions (S&C preceding or not in water training) on a one-day scale also appeared to be important for the S&C coaches regarding the optimization of the transfer (*n* = 8), as shown by some authors of a study on this topic (61). Responders suggested that overspeed mostly occurs after *in situ* resistance workout for a load contrast. This transfer could also take the form of an integrated workout including a part in dry-land and in swimming (i.e., alternated dry-land resistance exercises and swimming sprints, *n* = 4). For the responders to our survey, dry-land workouts alone (contrast training and / or strength endurance training) were not the best way to improve the quality of the transfer from dry-land to in-water conditions. These modalities are common practices aimed at improving sprint swimming performances and these topics were evaluated in the literature (62, 63). However, their effects on performance seem rather limited, and this can be explained by the fact that many factors may indeed influence the transfer (order between sessions, loads imposed on sessions). More specifically, the “S&C preceding swimming session” order was more cited than the “Swimming preceding S&C session” order and was intentionally chosen by the coach. From a scientific point of view, a review on this topic (64) showed that the long-term effects of concurrent S&C and swimming training were not impacted by the order between the sessions. A short (few minutes) recovery period between sessions also led to improved performance. On the other hand, the acute effects of one session had a negative impact on the next. Thus, if the main objective of the day is to create adaptations in the water, swimming sessions should be placed first.

These different results illustrate the coach's dilemma to take scientific evidence, environmental constraints and intended objectives in the training plans into account. Moreover, the identification of specific workouts that would improve the quality of the transfer from dry-land to swimming conditions remains unclear and would bridge the gap between science and the field.

### 4.3 Testing practices

Classic strength training exercises, in addition to being frequently prescribed, are also described as the most used in the literature for assessing dry-land strength levels on swimmers (65). It seems important that the scientific and field-testing practices are in adequacy (1). On-field testing practices is thus also an important process to investigate.

In dry-land specific conditions, some S&C coaches (*n* = 11) reported the use of force and velocity measurements in order to monitor the progression of these qualities. The accessibility—especially due to the price—to modern S&C evaluation devices could explain why some coaches don't integrate testing processes into their training programs. Indeed, only S&C coaches working in National Training Centers, which may be in more favorable positions, reported the use of expensive devices.

For 79.2% of responders (*n* = 19), it was commented that parallel observation of progress in dry-land condition and in-water condition was carried out. Indicators used to observe this progression were mainly the swimming performance (*n* = 10) and swimming spatio-temporal parameters: distance per cycle, number of strokes and stroke frequency (*n* = 4). Although this approach based on simple kinematic parameters is important, the coach's perception should also be supported by force and power evaluation in dry-land and in free swimming conditions, as well as by the estimation of propelling efficiency which illustrates the way the total mechanical power output is used beneficially to overcome body drag (66). In this aim, evaluation of force-velocity profiling both in dry-land and swimming conditions (67) might provide useful insights into the understanding of transfer optimization.

### 4.4 Limits

This study provided a detailed insight into what coaches are currently doing as regards to Strength and Conditioning with a focus on the transfer from dryland to in-water. However, some limitations should be recognized. First, although the survey used a rigorous construction process and conformed to the CHERRIES checklist (40), it should be noted that the questionnaire was not previously validated in the literature, which could have provided a different perspective on the validity of the questions in assessing the different variables. In the present case, it should be noted that the potential risks of bias linked to the use of a non-validated questionnaire remain reduced in view of the quantitative data collected and the choice of target population analysed. More qualitative data was also considered, such as task descriptions, types of workouts, and methodology of training, in a holistic approach of S&C in support of aquatic performance.

The sample size for this study was relatively small (24 coaches) compared to the estimated range of the coaching population in France. However, as the aim was to explore the practices of top-level coaches in terms of S&C in France, the representativeness in terms of top-level practice seems appropriate. Despite this, a larger sample at lower levels would enable the results of this study to be generalized to the entire coaching population. In the same philosophy, the sample of people surveyed in this study raises the question of international representativeness, given that S&C coaches questioned were exclusively French and that qualification and culture could differ from one country to another. It may also be of interest to bear in mind that the answers may have differed according to the specialty (stroke/distance) of the swimmers. However, as the great majority of S&C coaches trained sprinters, this limitation may be minimized. Finally, asking the S&C coaches questioned to differentiate their answers according to the gender/level/age of the swimmers would have deepened the discussions and is therefore an area of exploration for future studies.

## 5 Practical applications

This survey investigated the thoughts and practices on Strength and Conditioning from dryland and its transfer to in-water. Despite different practices from one coach to another, some trends in S&C training emerged and highlighted several possible practical applications:

- S&C coaches may refer and stay informed about the scientific literature to refine their field practices. The most significant gaps between coaches' perceptions and knowledge from the scientific literature seem to be related to 1. The order between dry-land and swimming session sequence. Responders would prefer to set the dry-land S&C session before the swimming session whereas scientific literature indicates no significant differences on long-term swimming performance whatever the order between the two sessions. 2. The use of resistance training in water with the aim of transferring abilities from dry-land. Indeed, a few scientific literatures investigated this aspect and some of them indicated that these common practices may have a limited impact on swimming performance.

- S&C coaches may develop the testing and monitoring of various parameters in both environments (i.e., dry-land and swimming) for a better understanding of transferring strength, power, and endurance abilities from dry-land to swimming. User-friendly and affordable resources such as mobile applications for S&C evaluation, and spatio-temporal analysis in swimming could provide valuable information. However, new emerging technologies such as electromechanical devices may also be of interest as regards to S&C evaluation.

## Data Availability

The raw data supporting the conclusions of this article will be made available by the authors, without undue reservation.
